# Work engagement among polish nurses: the mediating effects of personal resources and job crafting

**DOI:** 10.3389/fpubh.2026.1811994

**Published:** 2026-04-28

**Authors:** Grzegorz Wójcik, Michał Kulisz, Antoni Wontorczyk

**Affiliations:** Faculty of Management and Social Communication, Institute of Applied Psychology, Jagiellonian University, Kraków, Poland

**Keywords:** job crafting, nurses, POS, resiliency, self-efficacy, support at work, work engagement

## Abstract

**Background:**

The global shortage of nurses and their decrease in workplace-related wellbeing is a major public health challenge. It was intensified by the COVID-19 pandemic and is further driven by high job demands, lacking resources and reduced work engagement. Understanding organizational and individual factors that support nurses' engagement is therefore crucial.

**Objective:**

This study aims to examine how job resources - perceived organizational support and community support at work—relate to work engagement among nurses, and whether particular personal resources (self-efficacy and ego-resiliency) and job crafting mediate these relationships.

**Methods:**

To check the assumed effects, a cross-sectional survey was conducted among 270 female-only nurses employed at a large polish university hospital with 38 units and approximately 150 000 hospitalizations/year. Validated instruments were used to assess the degree of work engagement, perceived organizational support, community support at work, self-efficacy, ego-resiliency, and job crafting. Mediation analyses were conducted, controlling for relevant sociodemographic and occupational variables.

**Results:**

Perceived organizational support (POS) and community support at work were positively associated with work engagement when analyzed separately [for POS as the independent variable: B = 1.185, 95% CI = (0.872, 1.498), *t* = 7.457, *p* < 0.001; for community support as the independent variable: B = 1.108, 95% CI = (0.604, 1.612), *t* = 4.328, *p* < 0.001]; however, only POS remained a significant predictor when both resources were considered simultaneously [B = 1.074, 95% CI = (0.724, 1.423), *t* = 6.043, *p* < 0.001]. Mediation analyses indicated that self-efficacy, ego-resiliency, and job crafting partially mediated these relationships, with job crafting showing the strongest indirect effects, particularly for community support [B = 0.491, 95% CI = (0.262, 0.769)]. In more complex models including multiple mediators, indirect-only mediation patterns emerged, suggesting that job resources may influence work engagement primarily through personal and behavioral mechanisms rather than direct effects.

**Conclusion:**

The findings highlight the importance of supportive organizational environments and proactive work behaviors in enhancing nurses' engagement, emphasizing the need of interventions which strengthen leadership support, foster personal resources, and enable job crafting.

## Introduction

The global shortage of nurses, which has become even more severe after COVID-19 pandemic, is a serious public health problem as nurses play a key role in the provision of health services (1). One of the main factors influencing the global shortage is the high turnover rate of nurses related to exposure to stressful situations caused by the combination of high job demands and low job resources (2). An increase in nurse turnover will lead to many adverse effects on the healthcare system, such as a reduction in the number of experienced nurses and excessive workload for the remaining ones, a reduction in the level of medical safety of patients, as well as an increase in the incidence of falls and stress injuries (3), putting the implementation of United Nations' Sustainable Development Goal no. 3: Good Health and Wellbeing (4) under risk.

One of the main resources in the nursing profession is work engagement, defined as an affective-cognitive state of mind that can be characterized as a constant, deepening positive attitude toward professional responsibilities and people associated with work (5). This concept is categorized by vigor, i.e., extraordinary stages of dynamism; dedication, i.e., feeling of importance, passion, motivation; and absorption understood as a presence of completely strenuous and happily occupied with an individual's labor (5). Research indicates that higher work engagement among nurses is strongly correlated with low turnover intentions (6). A strong positive correlation has also been demonstrated between work engagement and the quality of care services, patient satisfaction, and job satisfaction among nurses (7, 8). Therefore, research on factors that influence increased work engagement in this professional group seems extremely important.

According to systematic reviews (7, 8), work engagement among nurses depends largely on factors at the institutional, organizational, and individual levels. Predictors of engagement at the institutional level include motivation, value convergence between the institution and the nurse, quality of materials, and organizational support; to those at the organizational level variables included work environment, rewards, ward climate, social context, sense of justice, sense of being part of a community, workload, and control. Individual-level predictors included optimism and a sense of professional efficacy. The important role of supervisors in building work engagement among nursing staff members was also highlighted. They should not only act as administrators, but as leaders caring for the wellbeing of subordinates.

According to Job Demands-Resources (JD-R) theory, job resources, such as support from supervisors and colleagues, feedback, development opportunities, stimulate work engagement and foster positive organizational outcomes, such as organizational commitment and better work performance through motivational processes (9). The relationship between organizational support and work engagement among nurses is well documented in nursing research. For example, in a study of 510 Australian and 718 American nurses, Brunetto et al. (10) demonstrated that perceived organizational support was related to work engagement in nurses from both countries. Similar findings were obtained in a study conducted among Malaysian nurses (11). However, research indicates that not only organizational support but also support derived from coworkers is related to greater work engagement. A meta-analysis conducted by Broetje et al. (12) demonstrated that support from supervisors and coworkers are key resources in nurses. In the case of Algerian nurses, Pohl et al. (13) indicated that both support from supervisors and coworkers have a positive impact on work engagement. Thus, we present hypothesis No. 1.

***H1:*** *Work engagement will be predicted by job resources i.e., perceived organizational support and community support at work separately and taken together*.

An extended JD-R model stated that personal resources could impact work engagement indirectly, pointing to their mediating role between job resources and work engagement (14). This mechanism, according to the Conservation of Resources Theory (COR), involves triggering a gain spiral, i.e., an individual's tendency to invest their resources whenever possible, thus increasing their pool of resources (15). Such resources may be of individual nature, e.g., as self-efficacy and ego-resiliency. Although both constructs are interrelated, as suggested by the fact that they were both included in the concept of psychological capital (16), there are significant differences between them. General self-efficacy, as the belief in successfully performing a task, is more closely related to task-focused coping, whereas ego-resiliency refers to adaptive flexibility in stressful situations while maintaining an internal sense of stability. In this approach, ego-resiliency is more related to emotion-focused coping and appropriate emotional regulation (17, 18).

In the context of the nursing profession, it has been shown that both self-efficacy (19) and ego-resiliency (20) are strong predictors of work engagement. Using the JD-R model, Pérez-Fuentes et al. (21) demonstrated that self-efficacy was the strongest predictor for all of the dimensions of work engagement among 1,777 Spanish nurses. In terms of ego-resilience, Cao and Chen (20) demonstrated that psychological resilience became the strongest predictor of work engagement, ahead of social support and empathy among nurses working in nephrology departments. In the context of JD-R theory, both self-efficacy and resilience could be developed through organizational enablers such as supportive supervision leading to employee engagement (16, 22). Wang et al. (16) have reported the mediating role of psychological capital (self-efficacy, optimism, hope, resilience) on the relationship between a perceived organizational support and engagement. Based on mentioned studies, we formulate hypothesis 2:

***H2:*** *Perceived organizational support (POS) and community support at work (CS) will indirectly affect work engagement via personal resources – self-efficacy (SE) and ego-resiliency (ER)*.

For clarity, we present the tested subhypotheses:

*H2.1. Self-efficacy mediates the POS*→*work engagement relationship*
*H2.2. Ego-resiliency mediates the POS*→*work engagement relationship*
*H2.3. SE mediates CS*→*work engagement relationship*
*H2.4. ER mediates CS*→*work engagement relationship*.
*H2.5. SE and ER mediate the POS and CS* ➔ work *engagement relationship*

Job crafting has recently received attention as a crucial factor affecting work engagement and was included in the extended JD-R model to explain motivational processes such as work engagement (23). According to Wrzesniewski and Dutton (24), the concept of job crafting refers to the process of improving and streamlining work, which takes place at the employee's initiative, often without the knowledge of supervisors. The authors indicate, however, that supervisors and colleagues play an important role in the opportunity to perform these types of behaviors. More recent studies highlighted that job crafting should not be considered as a private behavior but rather influenced by social context including the role of supervisor and supportive relationships at work (25). Regarding the nursing profession, previous research has shown that job crafting effectively impacts nurses' cognitive and emotional responses, leading to changes in task characteristics, interpersonal relationships, and ways of thinking about work, thereby enhancing their work engagement (26). Research conducted among Chinese nurses using the JD-R model confirmed that job crafting was associated with a higher level of commitment and job resources (16). In turn, the study by Park et al. (27) showed that individual resources in the form of psychological capital lead nurses to make job crafting adjustments they think are necessary, which in turn contributes to increased work engagement.

Current research among nurses confirms the presence of the effect of organizational support on work engagement through job crafting (28). Moreover, in qualitative research conducted among Chinese nurses, it was proven that not only organizational support, but also interpersonal relationships in the form of collaborative and inclusive team culture significantly influence job crafting behaviors (29). Since there is little research on the influence of the supervisor's and coworker's role on work engagement through job crafting behaviors among nurses, especially in the context of Central and Eastern Europe, in hypothesis 3 we assume that:

***H3:*** *Job crafting will mediate the positive relationship between job resources (perceived organizational support and community support at work) and work engagement in Polish nurses*.

Below we present subhypotheses:

*H3.1. JC mediates the POS* ➔ *work engagement relationship*
*H3.2. JC mediates the CS* ➔ *work engagement relationship*
*H3.3. JC with SE and ER mediate the POS and CS* ➔ *work engagement relationship*.

## Study objective

The study aims to examine how job resources—perceived organizational support and community support at the workplace—relate to work engagement among nurses, and whether particular personal resources (self-efficacy and ego-resiliency) and job crafting mediate these relationships.

## Material and methods

### Data collection

The cross-sectional study was carried out at all units of a large university hospital in Poland with 38 units, 1,288 beds and approximately 150 000 hospitalizations/year. At the time of the study conduction, 1,789 nurses were employed at the hospital.

The survey was presented in an online form (using Qualtrics software) to all nurses employed at the hospital. Data collection took place in January 2022 during the Omicron phase of the COVID-19 pandemic. At that time, high mortality and rapidly growing counts of infection cases could be registered, comparing to other periods of the pandemic in Poland. For example, on 29.12.2021 it reached its peak with 795 registered deaths (30) and 53,420 registered infection cases on 26.12.2021 (31).

### Ethical approval

The study received approval by the Jagiellonian University Bioethics Committee on the 15.12.2021 (decision no. 1072/6120/29/82021). All procedures followed relevant ethical guidelines and regulations.

### Participants and procedure

Eligibility criteria included: identifying as female and being professionally employed as a nurse. We decided to focus specifically on female as our aim was to unify the study sample. Male nurses represent only a small fraction of all employed nurses in most countries in the world, e.g., 11, 4% in the UK (32) and 2.9% in Poland (33). The survey was provided to all female nurses employed at the hospital. Before being presented the questionnaire, written informed consent was electronically obtained. Moreover, filling out the survey, respondents were notified about the general aims of the study, its voluntary and non-invasive nature and the anonymous processing of collected data. Participants were informed that they could resign at any time. Contact details of the researchers were provided to enable requesting additional information or clarification. The average time which the participants needed to fill out the questionnaire was 20 min. Finally, after completion of the study, the participants were compensated with gift cards.

A total number of 311 nurses (17.38% of the 1,789 employed at the hospital) completed the survey. After excluding 41 cases with incomplete responses or failure to meet the inclusion criteria, the final sample included 270 participants.

## Measures

### Work engagement

Work engagement was measured using the Utrecht Work Engagement Scale (UWES) developed by Schaufeli, Salanova, Goznales-Roma and Bakker (5) in the Polish adaptation by Szabowska-Walaszczyk, Zawadzka and Wojtaś (34). The scale contains 17 items measuring three indicators of work engagement – vigor (e.g., “At work, I feel bursting with energy”), dedication to work (e.g., “The work I do is meaningful and purposeful to me”) and absorption in work (e.g., “Time flies when I am working”). Responses are given on a seven-point scale, where 0 means ‘never' and 6 means “every day.” In this study, Cronbach's alpha coefficient was 0.93.

### Perceived organizational support and community support at work

Community support at work (CS) and perceived organizational support (POS) were measured using the Areas of Worklife Survey developed by Leiter and Maslach (35) in its Polish adaptation by Terelak and Izwantowska (36). The measurement of POS was based on two subscales—Rewards (consisting of four items) and Fairness (six items). According to the authors (36), the construct of perceived organizational support refers to employees' perceptions of receiving rewarding and fair behavior from the organization and its members. It should be noted that this definition puts emphasis on different aspects of this phenomenon than the most popular approach by Eisenberger and Stinglhamber (37), who define it as employees' general belief that their organization values their contribution and cares about their wellbeing. The Rewards subscale allows to study how workers perceive social and intrinsic rewards, while Fairness refers to the perceived equity and respectfulness of decision-making at the workplace. Scores from both subscales (Rewards and Fairness) were summed to create a perceived organizational support index. Organizational support in this view refers to more formal support channels, including (but not limited to) support from supervisors.

Community support at work was defined by the authors as an interpersonal interaction that involves emotional support and practical assistance and consists of receiving feedback on one's performance at work or an assessment (36, 38). From this perspective, the concept refers mostly to interactions within a team of employees. The construct was measured using the Community scale, consisting of 5 items referring to the perceived level of support received (e.g., ‘My team members support each other'). Responses for all subscales were given on a five-point scale ranging from 1 to 5 (“totally agree”). In our study all scales demonstrated acceptable internal consistency: Cronbach's alpha for community support at work was 0.87, while Cronbach's alpha for perceived organizational support (as a sum of two mentioned subscales) was 0.78.

### Self-efficacy

General self-efficacy was measured using the General Self-Efficacy Scale (GSES) (17) in its second version of the Polish adaptation (39). The scale consists of 10 statements (e.g., “I am always able to solve difficult problems if I try hard enough”) rated on a four-point scale from 1 (“no”) to 4 (“yes”). Reliability in this study was high (Cronbach's α = 0.88).

### Mental resiliency

Mental resiliency was assessed with the Polish version (40) of Ego-Resiliency Scale (ER-12) (18). The questionnaire includes 12 items describing adaptive, emotional and behavioral responses to stress (e.g., “I quickly recover and get over it when something surprises me”. Participants rated each statement on a four-point scale from 1 (“does not apply to me at all”) to 4 (“applies to me very strongly”). Cronbach's α obtained in the present sample was 0.84.

### Job crafting

Job crafting behaviors were measured using the Job Crafting Questionnaire (JCQ) (41) in its Polish adaptation (42). The scale consists of 15 items, allowing to measure three aspects of job crafting: task-related changes, cognitive reframing of work, and relational crafting. Responses were provided on a six-point scale ranging from 1 (“almost never”) to 6 (“very often”). The reliability of the scale was good (Cronbach's α = 0.86).

## Statistical analysis

Statistical analyses were conducted using IBM SPSS (version 31.0), Hayes' PROCESS macro version 5.0 and the R environment. The sequence of performing the analyses was as follows: We initially performed an analysis of descriptive statistics, which was followed by a check of assumptions of regression and mediation analysis. The normality of residuals was evaluated using the Shapiro–Wilk test for each linear model. Multicollinearity was assessed using tolerance values (>0.1) and variance inflation factors (VIF < 10). Autocorrelation of residuals was also checked for, using the Durbin–Watson statistic, which showed values close to 2.0, indicating no violation. Normality of residuals and heteroskedasticity were assessed optically. Finally, regression analysis using entering method, and further mediation analyses were performed using PROCESS Model 4 with a 95% confidence interval and 5,000 bootstrap resamples, recommended by Andrew F. Hayes (43).

All analyses were controlled for relevant sociodemographic variables, including workplace setting, years of work experience, education, marital status, and number of children. An indirect effect was interpreted as statistically significant when the confidence interval did not include zero.

The obtained effects were checked for power considering the research sample *post hoc* using G^*^power. All models tested within mediation analysis reached satisfactory levels (>0.80) (Table 1).

**Table 1:** Statistics of models tested within mediation analyses (*N* = 270).

Independent variable	Dependent variable	R	f^2^	MSE	F	*p*	Power (1- β)
POS	SE	0.275	0.082	12,664	2,113	0,024	0.996
POS + SE	UWES	0.511	0.353	199,148	8,271	0,000	1.000
POS	UWES	0.444	0.246	215,350	6,373	0,000	1.000
POS	RE	0.284	0.088	29,018	2,279	0,014	0.998
POS + RE	UWES	0.547	0.426	188,924	9,988	0,000	1.000
POS	JC	0.312	0.108	145,054	2,792	0,003	0.999
POS + JC	UWES	0.559	0.455	185,191	10,662	0,000	1.000
CS	JC	0.363	0.152	139,528	3,929	0,000	1.000
CS + JC	UWES	0.475	0.291	208,670	6,823	0,000	1.000
CS	UWES	0.303	0.101	243,717	2,616	0,005	0.999
POS + CS	SE	0.282	0.087	12.655	2.031	0.026	0.994
POS + CS	RE	0.292	0.093	28.991	2.187	0.016	0.996
POS + CS	JC	0.377	0.167	138.334	3.897	< 0.001	1.000
POS + CS + SE + RE	WE	0.560	0.477	186.420	8.985	< 0.001	1.000
POS + CS + SE + RE + JC	WE	0.605	0.577	172.928	10.493	< 0.001	1.000
POS + CS *(total effect)^a^*	WE	0.451	0.255	214.597	5.987	< 0.001	1.000

All models include the following covariates: work experience, workplace, education level, marital status, and number of children. The following abbreviations are used: MSE, mean square error; POS, perceived organizational support; CS, community support at work; SE, self-efficacy; RE, resiliency; WE, work engagement.

^a^Total effect of perceived organizational support/community support at work on work engagement.

## Results

### Sample characteristics

The study sample consisted of 270 participants. As presented in Table 2, all respondents were female, with a mean age of 37 years (SD = 12.2). Regarding parental status, the group was relatively evenly distributed: 51.1% of respondents reported having no children, whereas 37% indicated having one or two children. The majority of participants reported being in a relationship (71.5%), including both formal and informal partnerships. With respect to professional experience, 41.1% of the sample had at least 16 years of experience in the nursing profession, while approximately a quarter of respondents had worked as a nurse for no more than 2 years. Educational attainment was generally high, as most participants held a higher education degree (88%).

**Table 2:** Demographic characteristics of nurses.

Characteristics	*n* (%)
Gender	
*Female*	270 (100)
Relationship	
*Single*	77 (28.5)
*In relationship*	193 (71.5)
Number of children	
*0*	138 (51.1)
*1-2*	100 (37.0)
*3 and more*	32 (11.9)
Level of education	
*Higher (MA or PhD)*	127 (47.0)
*Higher (BA)*	111 (41.1)
*Secondary*	32 (11.9)
Work experience (years)	
*0-2*	69 (25.6)
*3-15*	90 (33.3)
*16 and more*	111 (41.1)
Workplace (hospital unit)	
*OP/ER*	36 (13.3)
*ICU*	53 (19.6)
*Other*	181 (67.0)

The following abbreviations are used: ICU, Intensive Care Unit; OP, Operating Block; ER, Emergency Room.

Descriptive statistics and intercorrelations among the study variables are presented in Table 3. The results (for consistency all presented as mean raw scores) indicate that participants reported moderate levels of work engagement (M = 62.90; SD = 16.07). Regarding the mediating variables, job crafting reached a moderate value (M = 53.78; SD = 12.71), resiliency was slightly below moderate (M = 35.41; SD = 5.51), whereas self-efficacy was relatively high (M = 30.38; SD = 3.63). Finally, average levels of community support at work could be observed (M = 18.06; SD = 3.71), whereas perceived organizational support was relatively high (M = 30.64; SD = 5.62). Correlation analyses revealed that all associations between the examined variables were statistically significant. It should be acknowledged that some of the variables displayed weak bivariate correlations (e.g., community support at work with self-efficacy: *r* = 0.142, *p* < 0.05 or with resiliency: *r* = 0.159, *p* < 0.05). Therefore, the further described relationships deserve cautious interpretation.

**Table 3:** Means, standard deviations, and pearson's coefficient correlations between job demands and burnout (*N* = 270).

Variable	Mean	SD	1a	1b	1c	1d	1e	1f
1a. Work engagement	62.90	16.07	–	0.419^**^	0.418^**^	0.326^**^	0.256^**^	0.415^**^
1b. Resiliency	35.41	5.51	–	–	0.362^**^	0.504^**^	0.159^*^	0.224^**^
1c. Job crafting	53.78	12.71	–	–	–	0.302^**^	0.277^**^	0.207^**^
1d. Self–efficacy	30.38	3.63	–	–	–	–	0.142^*^	0.197^**^
1e. Community support at work	18.06	3.71	–	–	–	–	–	0.450^**^
1f. Perceived organizational support	30.64	5.62	–	–	–	–	–	–

Statistical significance is indicated as follows:

^*^p < 0.05;

^**^p < 0.001.

### Hypotheses testing

**Hypothesis 1** proposed that job resources—perceived organizational support (POS) and community support at work (CS)—would positively and directly predict work engagement (WE), both with POS and CM included together in a model, as well as separately. As shown in Table 4, the hypothesis can be partially confirmed. The job resources were in a direct association with work engagement separately [for POS as the independent variable: B = 1.185, 95% CI = (0.872, 1.498), *t* = 7.457, *p* < 0.001; for community support at work as the independent variable: B = 1.108, 95% CI = (0.604, 1.612), *t* = 4.328, *p* < 0.001]. However, if the two resources were analyzed in a model together, only POS entered the model [for POS as the independent variable: B = 1.074, 95% CI = (0.724, 1.423), *t* = 6.043, *p* < 0.001; for community support at work as the independent variable: B = 0.375, 95% CI = (−0.156, 0.905), *t* = 1.391, *p* = 0.165].

**Table 4:** Regression analysis of perceived organizational support (POS) and community support at work (CS) as predictors of work engagement (WE) (*N* = 270).

Variables tested inside the joint model	Unstandardized coefficient (B)	Standardized coefficient (β)	Standard Error (SE)	*t*	*p*	95,0% CI for B	
						LL	UL
POS	1.074	0.376	0.178	6.043	< 0.001	0.724	1.423
CS	0.375	0.086	0.269	1.391	0.165	−0.156	0.905
**Variables tested separately**	**Unstandardized coefficient (B)**	**Standardized coefficient (**β**)**	**Standard Error (SE)**	***t***	***p***	**95,0% CI for B**	
						**LL**	**UL**
POS	1.185	0.415	0.159	7.457	< 0,001	0.872	1.498
CS	1.108	0.256	0.256	4.328	< 0,001	0.604	1.612

The following abbreviations are used: LL, the lowest value of the confidence interval; UL, the highest value of the confidence interval; POS, perceived organizational support; CS, community support at work.

**Hypothesis 2** assumed indirect associations between POS and CM with work engagement through two mediating personal resources (self-efficacy and ego-resiliency). This hypothesis can be partly confirmed, with four subhypotheses confirmed and one partially confirmed.

As stated in subhypothesis 2.1, self-efficacy would mediate the relationship between POS and work engagement. As shown in Table 5, POS was positively related to work engagement [B = 1.237, 95% CI = (0.901, 1.572), *t* = 7.259, *p* < 0.001]. After including self-efficacy and the control variables in the model, the relationship between work engagement and POS remained significant [B = 1.085, 95% CI = (0.756, 1.414), *t* = 6.494, *p* < 0.001], suggesting a partial mediation. The bootstrapped standardized indirect effect between POS and work engagement, accounted for by self-efficacy, differed significantly from zero [B = 0.152, 95% CI = (0.043, 0.321)].

**Table 5:** Mediating effect of self-efficacy (SE) in association between perceived organizational support (POS) and work engagement (WE) (*N* = 270).

Tested relationship	Unstandardized coefficient (B)	Standardized coefficient (β)	Standard error (SE)	*t*	*p*	LLCI	ULCI
POS → WE (total effect)^a^	1.237	0.433 (0.204)	0.170	7.260	< 0.001	0.901	1.572
POS → SE	0.132	0.204 (0.039)	0.041	3.182	0.002	0.050	0.213
SE → WE	1.158	0.262 (0.086)	0.246	4.698	< 0.001	0.672	1.643
POS → WE (direct effect)^b^	1.085	0.380 (0.164)	0.167	6.494	< 0.001	0.756	1.414
POS → SE → WE	0.152	0.053	0.072			0.043	0.321

The model includes the following covariates: work experience, workplace, education level, marital status, and number of children. The following abbreviations are used: LLCI, the lowest value of the confidence interval; ULCI, the highest value of the confidence interval; POS, perceived organizational support; SE, self-efficacy; WE, work engagement.

^a^Total effect of perceived organizational support on work engagement.

^b^Direct effect of perceived organizational support on work engagement after including self-efficacy as a mediator.

A similar pattern could be found for ego-resiliency, as the variable accounting for the relationship between POS and work engagement (Table 6). This should support the claims of subhypothesis 2.2. Like in the previous described model, POS showed a significant positive relationship with work engagement. When ego-resiliency and covariates were entered into the model, the relationship between POS and work engagement remained significant [B = 1.007, 95% CI = (0.684, 1.330), *t* = 6.140, *p* < 0.001], which might indicate partial mediation. Bootstrapping analyses revealed a significant indirect relationship between POS and work engagement, accounted for by resiliency [B = 0.230, 95% CI = (0.101, 0.388)]. The mediating effect of resiliency in the relation between POS and work engagement was 18.65% (0.2301/1.2369), remaining stronger than the effect of self-efficacy: 12.30% (0.1522/1.2369).

**Table 6:** Mediating effect of resiliency (RE) in association between perceived organizational support (POS) and work engagement (WE) (*N* = 270).

Tested relationship	Unstandardized coefficient (B)	Standardized coefficient (β)	Standard error (SE)	*t*	*p*	LLCI	ULCI
POS → WE (total effect)^a^	1.237	0.433 (0.204)	0.170	7.260	< 0.001	0.901	1.572
POS → RE	0.238	0.243 (0.056)	0.063	3.803	< 0.001	0.115	0.361
RE → WE	0.967	0.332 (0.144)	0.159	6.101	< 0.001	0.655	1.280
POS → WE (direct effect)^b^	1.007	0.352 (0.146)	0.164	6.140	< 0.001	0.684	1.330
POS → RE → WE	0.230	0.081	0.073			0.101	0.388

The model includes the following covariates: work experience, workplace, education level, marital status, and number of children. The following abbreviations are used: LLCI, the lowest value of the confidence interval; ULCI, the highest value of the confidence interval; POS, perceived organizational support; RE, resiliency; WE, work engagement.

^a^Total effect of perceived organizational support on work engagement.

^b^Direct effect of perceived organizational support on work engagement after including resiliency as a mediator.

In subhypothesis 2.3 it was assumed that self-efficacy would mediate the relationship between community support at work and work engagement. The results (Table 7) suggest that this assumption might be correct as the two latter variables were positively associated [B = 1.063, 95% CI = (0.546, 1.580), *t* = 4.052, *p* < 0.001]. After including self-efficacy and the control variables in the model, the relationship between community support at work and work engagement remained significant [B = 0.876, 95% CI = (0.379, 1.374), *t* = 3.468, *p* < 0.001], which could indicate partial mediation. The bootstrapped standardized indirect association between POS and work engagement, accounted for by self-efficacy, differed significantly from zero [B = 0.187, 95% CI = (0.015, 0.418)].

**Table 7:** Mediating effect of self-efficacy (SE) in association between community support at work (CS) and work engagement (WE)—first measurement (*N* = 270).

Tested relationship	Unstandardized coefficient (B)	Standardized coefficient (β)	Standard error (SE)	*t*	*p*	LLCI	ULCI
CS → WE *(total effect)*^*a*^	1.063	0.245 (0.063)	0.262	4.052	< 0.001	0.546	1.580
CS → SE	0.139	0.142 (0.021)	0.060	2.310	0.022	0.021	0.258
SE → WE	1.341	0.303 (0.105)	0.258	5.209	< 0.001	0.843	1.848
CS → WE *(direct effect)^*b*^*	0.876	0.202 (0.047)	0.253	3.468	< 0.001	0.379	1.374
CS → SE → WE	0.187	0.043	0.102			0.015	0.418

The model includes the following covariates: work experience, workplace, education level, marital status, and number of children. The following abbreviations are used: LLCI, the lowest value of the confidence interval; ULCI, the highest value of the confidence interval; CS, community support at work; SE, self-efficacy; WE, work engagement.

^a^Total effect of community support at work on work engagement.

^b^Direct effect of community support at work on work engagement after including self-efficacy as a mediator.

Similarly, in the subhypothesis 2.4, we assumed that the relationship between community support at work and work engagement would be mediated by resiliency. As suggested by the results presented in Table 8, this subhypothesis should be confirmed as community support at work and work engagement were in a positive relationship (as mentioned in the description of the previous model), which remained significant [B = 0.801, 95% CI = (0.318, 1.284), *t* = 3.264, *p* = 0.001] after adding resiliency as a potential mediator and control variables to the model. The bootstrapped standardized indirect association between community support at work and work engagement, accounted for by resiliency, differed significantly from zero [B = 0.262, 95% CI = (0.040, 0.501)]. Similarly to the previous described model, these results indicate partial mediation. It should be added that the effect of resiliency in the relation between community support at work and work engagement was 24.64% (0.2619/1.0630), remaining stronger than the effect of self-efficacy: 17.58% (0.1869/1.0630).

**Table 8:** Mediating effect of resiliency (RE) in association between community support at work (CS) and work engagement (WE)—first measurement (*N* = 270).

Tested relationship	Unstandardized coefficient (B)	Standardized coefficient (β)	Standard error (SE)	*t*	*p*	LLCI	ULCI
CS → WE *(total effect)^*a*^*	1.063	0.245 (0.063)	0.262	4.052	< 0.001	0.546	1.580
CS → RE	0.237	0.159 (0.026)	0.092	2.577	0.011	0.056	0.418
RE → WE	1.107	0.380 (0.177)	0.164	6.750	< 0.001	0.784	1.429
CS → WE *(direct effect)^*b*^*	0.801	0.185 (0.041)	0.245	3.264	0.001	0.318	1.284
CS → RE → WE	0.262	0.115	0.060			0.040	0.501

The model includes the following covariates: work experience, workplace, education level, marital status, and number of children. The following abbreviations are used: LLCI, the lowest value of the confidence interval; ULCI -the highest value of the confidence interval; CS, community support at work; RE, resiliency; WE, work engagement.

^a^Total effect of community support at work on work engagement.

^b^Direct effect of community support at work on work engagement after including resiliency as a media.

Finally, subhypothesis 2.5 stated that self-efficacy and ego-resiliency would mediate the relationships between both POS, community support at work and work engagement. The results indicate that this assumption was partly confirmed, as only relationships with POS were found significant. As shown in Table 9, POS was positively related to work engagement [B = 1.128, 95% CI = (0.758, 1.497), *t* = 6.012, *p* < 0.001]. After including resiliency and self-efficacy, as well as the control variables in the model, the relationship between work engagement and POS remained significant [B = 0.906, 95% CI = (0.555, 1.257), *t* = 5.084, *p* < 0.001], which might indicate partial mediation. The bootstrapped standardized indirect association between POS and work engagement, accounted for by resiliency, differed significantly from zero [B = 0.159, 95% CI = (0.032, 0.311)], similarly to the effect accounted for by self-efficacy [B = 0.063, 95% CI = (0.001, 0.193)]. The difference in the effect of resiliency accounting for the relation between POS and work engagement was 14.07% (0.1587/1.1277), being stronger than the effect of self-efficacy, which equaled 5.59% (0.0630/1.1277).

**Table 9:** Mediating effect of self-efficacy (SE) and resiliency (RE) in associations between perceived organizational support (POS), community support at work (CS) and work engagement (WE) (*N* = 270).

Tested relationship	Unstandardized coefficient B	Standardized coefficients β (f^2^)	Standard error	*t*	*p*	LLCI	ULCI
POS → WE *(total effect)^*a*^*	1.128	0.395 (0.140)	0.188	6.012	< 0.001	0.758	1.497
CS → WE *(total effect)^*a*^*	0.375	0.087 (0.007)	0.272	1.382	0.168	−0.160	0.910
POS → SE	0.111	0.046 (0.023)	0.046	2.426	0.016	0.021	0.200
POS → RE	0.205	0.210 (0.034)	0.069	2.979	0.003	0.070	0.341
CS → SE	0.072	0.074 (0.005)	0.066	1.091	0.276	−0.058	0.202
CS → RE	0.111	0.075 (0.005)	0.100	1.116	0.266	−0.085	0.308
SE → WE	0.570	0.129 (0.017)	0.274	2.084	0.038	0.032	1.109
RE → WE	0.773	0.256 (0.072)	0.181	4.276	< 0.001	0.417	1.128
POS → WE *(direct effect)^*b*^*	0.906	0.317 (0.101)	0.178	5.084	< 0.001	0.555	1.257
CS → WE *(direct effect)^*b*^*	0.248	0.057 (0.004)	0.254	0.977	0.330	−0.252	0.748
POS → SE → WE	0.063	0.022	0.051			0.001	0.193
POS → RE → WE	0.159	0.056	0.072			0.032	0.311
CS → SE → WE	0.041	0.010	0.052			−0.049	0.164
CS → RE → WE	0.086	0.020	0.090			−0.079	0.274

The model includes the following covariates: work experience, workplace, education level, marital status, and number of children. The following abbreviations are used: LLCI, the lowest value of the confidence interval; ULCI -the highest value of the confidence interval; POS, perceived organizational support; CS, community support at work; SE, self-efficacy; RE – resiliency; JC, job crafting WE, work engagement.

^a^Total effect of community support at work on work engagement.

^b^Direct effect of community support at work on work engagement after including resiliency as a mediator.

**Hypothesis 3** stated that job crafting would function as a mediator in the positive relationship between job resources (community support at work and perceived organizational support—POS) and work engagement. The hypothesis can be partially confirmed as all of its subhypotheses have been fully or partially confirmed.

In subhypothesis 3.1 it was stated that job crafting would mediate the relationship between POS and work engagement. This assumption could be confirmed. Job crafting significantly accounted for the associations between POS and work engagement. Moreover, as presented in Table 10, when job crafting with control variables were included in the analysis the relationship between POS and work engagement remained significant [β = 0.351, 95% CI = (0.685, 1.323), *t* = 6.200, *p* < 0.001], which might suggest partial mediation. The bootstrapped effect of the relationship between POS and work engagement, accounted for by job crafting, was statistically significant [β = 0.082, 95% CI = (0.097, 0.390)].

**Table 10:** Mediating effect of job crafting (JC) in association between perceived organizational support (POS) and work engagement (WE) (*n* = 270).

Tested relationship	Unstandardized coefficient (B)	Standardized coefficient (β)	Standard error (SE)	*t*	*p*	LLCI	ULCI
POS → WE (total effect)^a^	1.237	0.433 (0.204)	0.170	7.260	< 0.001	0.901	1.572
POS → JC	0.505	0.228 (0.050)	0.140	3.610	< 0.001	0.230	0.780
JC → WE	0.461	0.357 (0.167)	0.070	6.571	< 0.001	0.323	0.600
POS → WE (direct effect)^b^	1.004	0.351 (0.149)	0.162	6.200	< 0.001	0.685	1.323
POS → JC → WE	0.233	0.082	0.075			0.097	0.390

The model includes the following covariates: work experience, workplace, education level, marital status, and number of children. The following abbreviations are used: LLCI, the lowest value of the confidence interval; ULCI -the highest value of the confidence interval; POS, perceived organizational support; JC, job crafting; WE, work engagement.

^a^Total effect of perceived organizational support on work engagement.

^b^Direct effect of perceived organizational support on work engagement after including job crafting as a mediator.

A similar pattern was observed for community support at work (Table 11), which was significantly related to work engagement [β = 0.245, 95% CI = (0.546, 1.580), *t* = 4.052, *p* < 0.001]. This association stayed significant after adding job crafting as a potential mediator [β = 0.132, 95% CI = (0.073, 1.072), *t* = 2.255, *p* < 0.05]. Bootstrapping indicated an indirect effect [B = 0.491, 95% CI = (0.262, 0.769)] through job crafting. These results indicate that the subhypothesis 2.2, stating that job crafting would mediate the relationship between community support at work and work engagement, should be confirmed. The effect of job crafting in the relation between community support at work and work engagement was 46.18% (0.4909/1.0630), remaining stronger than its effect in the relationship between POS and work engagement: 18.83% (0.2329/1.2369) and all other tested models.

**Table 11:** Mediating effect of job crafting (JC) in association between community support at work (CS) and work engagement (WE) (*N* = 270).

Tested relationship	Unstandardized coefficient (B)	Standardized coefficient (β)	Standard error (SE)	*t*	*p*	LLCI	ULCI
CS → WE (total effect)^a^	1.063	0.245 (0.063)	0.262	4.052	< 0.001	0.546	1.580
CS → JC	0.970	0.289 (0.092)	0.199	4.879	< 0.001	0.578	1.359
JC → WE	0.507	0.392 (0.173)	0.076	6.671	< 0.001	0.357	0.657
CS → WE (direct effect)^b^	0.572	0.132 (0.020)	0.254	2.255	0.025	0.073	1.072
CS → JC → WE	0.491	0.113	0.130			0.262	0.769

The model includes the following covariates: work experience, workplace, education level, marital status, and number of children. The following abbreviations are used: LLCI, the lowest value of the confidence interval; ULCI -the highest value of the confidence interval; CS, community support at work; JC, job crafting; WE, work engagement.

^a^Total effect of community support at work on work engagement.

^b^Direct effect of community support at work on work engagement after including job crafting as a mediator.

Finally, subhypothesis 3.3 stated that job crafting, together with self-efficacy and resiliency would mediate the relationships between POS and work engagement, as well as between community support at work and work engagement. This assumption was partially supported, as significant indirect effects were observed, while the direct effects were not significant, indicating an indirect-only mediation pattern. Community support at work showed no significant relationship with work engagement [B = 0.375, 95% CI = (−0.160, 0.910), t = 1.382, *p* = 0.168]. The bootstrapped indirect effect of the relationship between POS and work engagement, accounted for by job crafting, was statistically significant [B = 0.274, 95% CI = (0.103, 0.501)]. As presented in Table 12, when job crafting, self-efficacy and resiliency with control variables were included in the analysis the direct association between POS and work engagement remained insignificant [B = 0.008, 95% CI = (−0.485, 0.500), *t* = 0.031, *p* = 0.976], which might indicate mediation based only on the indirect effect.

**Table 12:** Mediating effect of self-efficacy (SE), resiliency (RE) and Job crafting (JC) in associations between perceived organizational support (POS), community support at work (CS) and work engagement (WE) (*N* = 270).

Tested relationship	Unstandardized coefficient B	Standardized coefficients β (f^2^)	Standard error	*t*	*p*	LLCI	ULCI
POS → WE *(total effect)^*a*^*	1.128	0.395 (0.140)	0.188	6.012	< 0.001	0.758	1.497
CS → WE *(total effect)^*a*^*	0.375	0.087 (0.007)	0.272	1.382	0.168	−0.160	0.910
POS → SE	0.111	0.171 (0.023)	0.046	2.426	0.016	0.021	0.200
POS → RE	0.205	0.210 (0.034)	0.069	2.979	0.003	0.070	0.341
POS → JC	0.271	0.123 (0.013)	0.151	1.799	0.073	−0.026	0.567
CS → SE	0.072	0.074 (0.005)	0.066	1.091	0.276	−0.058	0.202
CS → RE	0.111	0.075 (0.005)	0.100	1.116	0.266	−0.085	0.308
CS → JC	0.803	0.240 (0.053)	0.218	3.685	< 0.001	0.374	1.233
SE → WE	0.392	0.089 (0.009)	0.266	1.473	0.142	−0.132	0.916
RE → WE	0.588	0.202 (0.043)	0.179	3.294	0.001	0.237	0.940
JC → WE	0.341	0.267 (0.082)	0.074	4.580	< 0.001	0.194	0.487
POS → WE *(direct effect)^*b*^*	0.871	0.305 (0.101)	0.172	5.071	< 0.001	0.533	1.210
CS → WE *(direct effect)^*b*^*	0.008	0.002 (0.000)	0.250	0.031	0.976	−0.485	0.500
POS → SE → WE	0.043	0.015	0.043			−0.007	0.154
POS → RE → WE	0.121	0.042	0.059			0.021	0.249
POS → JC → WE	0.092	0.020	0.057			−0.014	0.208
CS → SE → WE	0.028	0.007	0.042			−0.040	0.129
CS → RE → WE	0.066	0.015	0.072			−0.070	0.224
CS → JC → WE	0.274	0.063	0.101			0.103	0.501

The model includes the following covariates: work experience, workplace, education level, marital status, and number of children. The following abbreviations are used: LLCI, the lowest value of the confidence interval; ULCI, the highest value of the confidence interval; POS, perceived organizational support; CS, community support at work; SE, self-efficacy; RE, resiliency; JC, job crafting WE, work engagement.

^a^Total effect of perceived organizational support/community support at work on work engagement.

^b^Direct effect of perceived organizational support/community support at work on work engagement after including a mediator.

## Discussion

Work engagement among nurses plays a pivotal role in the development of organizational commitment, improving nursing performance, and the desire to remain in the profession (7, 8). Therefore, identifying the factors that determine work engagement in this professional group seems crucial. Our main contribution to the work engagement literature was to demonstrate how social support derived from the organization, i.e., supervisors and coworkers is related to work engagement through personal resources (self-efficacy and ego-resiliency) and job crafting in the group of Polish nurses. As evidenced by the existing literature, there is a lack of research on work engagement among Polish nurses, although engaged health professionals provide high-quality care, perform tasks beyond their duties, and, above all, decide to remain in the profession, which is a remedy for the high turnover intention rate in the analyzed professional group in Poland (44).

Regarding the pandemic context of our research, nurses' work engagement, including its latest phase, varied from moderate to high due to higher teamwork, greater moral commitment and strong professional meaning while after the pandemic era, the mean level of work engagement of health professionals, including nurses, turned out to be lower (45, 46). Our results confirm these findings as relatively high level of work engagement among nurses reported in this study has taken place during dramatic social situation of the latest wave of COVID-19 pandemic in Poland during which a huge number of deaths were recorded (30). The lower mean level of work engagement among nurses in the post-pandemic period can be explained by staff shortages and insufficient support for nurses from nursing leaders (47).

With respect to Hypothesis 1, we analyzed the relationships between job resources, such as perceived organizational support and community support at work, and work engagement among Polish nurses. With this assumption, we referred to the key claim of the JD-R theory, which highlights the role of job resources in the development of engagement, and consequently, the development of organizational commitment, initiative, and improved professional performance (41). Hypothesis 1 was partially confirmed. When analyzed separately, perceived organizational support and support received from community was found to be significant predictors of work engagement. These findings are consistent with previous studies. For example, Nasurdin et al. (48) and Mehrad et al. (49) have demonstrated that perceived organizational support and that derived from coworkers directly and positively influence work engagement among nurses. The importance of both organizational support and support of the workplace community for the development of work engagement can be explained using social exchange theory (50). When nurses perceive their organizations as supportive, caring for their needs and emotions, and providing feedback, they feel obligated to “reciprocate” by becoming more engaged in their duties. As a result, they develop greater affective organizational commitment and a desire to stay in the workplace.

Interestingly, contrary to the assumptions of Hypothesis 1.3, support received from one's workplace community was not a predictor of work engagement when both organizational support and community support were taken into account. This result could mean that perceived organizational support is the more robust direct predictor in the model compared with community support. For example, in a study by Othman and Nasurdin (11) coworker support, contrary to supervisor support, was not found to be a significant predictor of work engagement. The authors suggest that the lack of this relationship is caused by the large number and diversity of responsibilities of modern nurses, who lack time to establish closer contacts with their team members and their workplace social community, which could potentially influence the development of their professional engagement. This phenomenon might have strengthened during the COVID-19 pandemic as the workload might have gotten even more intense (45). Nurses may have remained more committed to their work when they perceived that they were supported by hospital management during such stressful time. Alternatively, community support may become less important than organizational support under the influence of high workload resulting from the pandemic and not be a predictor of work engagement in such case.

In Hypothesis No. 2, we assumed a mediating effect of self-efficacy and psychological resiliency between perceived organizational support and community support at work as well as work engagement. When the factors were analyzed in models including only one independent variable and only one mediator, all relationships between both kind of support and work engagement, as well as indirect relationships through self-efficacy and resiliency have been found significant. However, when both types of support at work—received from community and organization—as well as both personal resources were considered, it turned out that a significant relation occurs only in the relationship between perceived organizational support and work engagement, accounted for by resiliency. This suggests that, among nurses, work engagement is related to the perception of being supported by the hospital, as well as to emotional regulation and adaptive recovery under prolonged stress exposure. Higher levels of organizational support are in turn related to higher levels of ego-resiliency. As already shown, ego-resiliency is a crucial antecedent of work engagement among nurses (51). The obtained results suggest the importance of nursing supervisors fostering ego-resilience among their subordinates. It can be concluded that appropriate emotional regulation and effective emotion-focused coping, supported by the appropriate attitude of nursing managers, are crucial for increasing nurses' work engagement, one manifestation of which is a sense of emotional connection with the workplace.

In the context of the JD-R theory, the mediating effect of self-efficacy was demonstrated by Xanthopoulou et al. (52) in the relationship between job resources (autonomy, social support, support from the supervisor, professional development opportunities) and work engagement. As the authors explain, a higher level of perceived resources in the workplace activates a sense of self-efficacy, which leads to a belief in greater control in one's professional environment and, consequently, pride in one's work and engagement. The present findings are consistent with those of Pan et al. (22), who, in a study of Chinese nurses, demonstrated that psychological capital—comprising hope, resilience, self-efficacy, and optimism—mediated the relationship between a supportive work environment and nurses' work engagement.

In hypothesis 3 we assumed a mediating effect of job crafting in the relationship between community support at work, perceived organizational support, and work engagement. Our results suggest a mediating effect of job crafting on the relation between both POS and community support at work and work engagement when both kinds of support are analyzed separately. However, when both kinds of support were included in the model, only the indirect relationship between POS and work engagement through job crafting remained significant.

As demonstrated in prior studies, job crafting, defined as the process of shaping one's job in the form of physical and cognitive adjustments to work tasks, is related to greater work engagement and more positive affective states (27), as well as to others'– including colleagues and supervisors—behavior (24). It might therefore be an indispensable factor in explaining the relationship between community support at work and work engagement. In the nursing community, job crafting is often influenced by the quality of interactions, advice, respect, and assistance, which constitute external resources such as autonomy and work satisfaction (53). The influence of social variables on job crafting behaviors in this professional group can be explained by referring to the self-determination theory which emphasizes the importance of the social context in meeting basic psychological needs (54). In this perspective, nurses who feel appreciated by their superiors for their competence and are granted the necessary autonomy in performing tasks, are more likely to make the job-crafting adjustments which, as they believe, are necessary. Furthermore, with support from other nurses, they may feel they are not left alone with the problems they experience, and that their actions and ideas for improving their work gain the approval of their colleagues (relatedness), which encourages them to make further professional changes and is associated with a higher level of work engagement. Our results are consistent with Sheehan et al.'s study (25), which demonstrated a mediating effect of job crafting behavior in the relationship between high involvement work practices and work engagement among Australian nurses. Supervisor support, in turn, moderated the relationship between high involvement practices and job crafting, indicating that it was stronger when supervisor support was greater. Furthermore, research conducted among Chinese nurses showed that job crafting contributes to improving work relationships, promoting personal growth and wellbeing, which in turn translates into greater work engagement (55). As these results are corresponding with our results, it can be concluded that the relationship between job crafting and support from one's workplace community (coworkers and supervisors) is dual in nature: engaging in job crafting behaviors is positively related to work relationships, and positive work relationships are related to job crafting behaviors, which in both cases are connected with greater work engagement.

Interestingly, the effect of job crafting on the relation between community support at work and work engagement, was greater than the one on the association between POS and work engagement. This might mean that nurses are more engaged in job crafting activities focusing, e.g., on task characteristics, interpersonal relations, and ways of thinking about work when they feel that other coworkers and supervisors provide them with greater support, which is related to a higher level of job engagement. According to Tims and Parker (55) a supportive coworker environment increases collaborative job crafting, which takes the form of employees deciding how their work is organized and conducted together with each other. This in turn increases the level of work engagement of an individual. Therefore, it can be concluded that supportive relationships with other coworkers are more important for the development of job crafting activities than support received from organization.

## Limitations and practical implications

Although our study is one of the first to address the issue of the relationship between support from supervisors and co-workers, job crafting and work engagement in Poland, especially in the medical sector, several limitations should be outlined.

Firstly, the study is cross-sectional in nature, which prevents the identification of causal relationships. Thus, temporal sequencing is not established and therefore reverse causality might be possible. It would be therefore beneficial to conduct subsequent measurements on the same group of nurses to more strongly confirm the hypothesis of a causal relationship between job crafting behaviors and work engagement. Secondly, we considered only the aggregated job crafting indicator, but it would be interesting to additionally analyze the individual dimensions of this variable—task, relationships, and cognitive crafting—to determine whether and to what extent support received from workplace community and superiors are associated with the individual dimensions of the analyzed variable. Thirdly, the study included exclusively female nurses from a single hospital in a large city, which may limit the generalizability of the results to the population of Polish nurses, including also male nurses and those working in smaller towns. However, as we indicated earlier, the nursing profession is feminized worldwide, and in Poland, this feminization is particularly evident. In this context, the significant disparity between women and men in the analyzed sample would pose a methodological and statistical problem. Including only women also stems from the desire to standardize the study sample in terms of the analyzed variables due to existing gender differences and preferences. Another possible constraint on generalizability might be the fact that the study was conducted during Omicron surge in January 2022. Fourthly, all data have been collected through one questionnaire based on one software which may have contributed to developing common method bias. The questionnaire relied on self-report measures which might have encouraged the respondents to provide socially desirable responses. Another limitation is the small number of independent variables—job resources—analyzed in relation to job crafting behaviors and work engagement. Considering variables such as information sharing and employee participation, job security, performance management and career capital would help to determine more precisely which job resources contribute to greater work engagement among nurses through job crafting behaviors. Similarly, in addition to work engagement, other constructs such as turnover or patient satisfaction might be measured to explore the results of the functioning of the mentioned factors. Additionally, future directions should include intervention testing. For example, such an intervention could involve assigning nurses from the analyzed hospital to an experimental group that would participate in a month-long job crafting training program and a control group that would not. After some time, a real-world measurement would be conducted to determine whether the experimental group exhibited more job crafting behaviors and, at the same time, a higher level of work engagement compared to the control group.

## Practical implications

The obtained results suggest the importance of the role of supervisors and community at the workplace. As mentioned by Shalev et al. (56), supervisors should focus on organizing multidisciplinary meetings aimed at helping nurses identify negative emotions, correct misconceptions in order to increase satisfaction and work engagement among nurses. In this context, Ibrahim et al. (57) point out that policies that empower nurses to modify their tasks, relationships, and cognitive aspects of work have a positive impact on wellbeing and team cohesion, which will contribute not only to higher quality of care but also to reducing burnout among nurses. For example, in order to support job crafting behaviors and work engagement, support from coworkers and supervisors could, at the individual level, rely on shaping strategies such as joint discussion of innovative ways of organizing work, positive reframing, goal setting and problem solving, which contribute to increasing psychological capital, and at the organizational level, allowing nurses to make decisions at the ward level in the field of patient care activities or scheduling (28). In this context, it is essential to make shared decisions on assignment planning among nurses to ensure that it aligns with their preferences, competencies, and professional qualifications, while also maintaining equitable workload distribution and preventing excessive burden for specific team members. Furthermore, jointly agreed-upon shift scheduling that considers nurses' personal needs and work–life balance, as well as collaborative overtime planning within wards, can strengthen nurses' perceptions of fairness and equal treatment by their supervisors (47).

It is worth adding that, in the post-pandemic era, nursing managers should be aware of barriers experienced by their nurse subordinates in the form of staff shortages and high workload, which translate into limited job resources and decreased work engagement. In order to overcome these barriers, Aungsuroch et al. (47) emphasize the need to act from an organizational perspective by creating work climate emphasizing nurses' growth and career development, introducing participative or transformational leadership, organizational justice, workload distribution, job fit as well as developing assertive communication. Furthermore, Ojala et al. (58) propose developing effective and supportive relationships with supervisors and colleagues, which reduce anxiety, stress and intent to leave among newly graduated nurses, and on the other hand, increase self-confidence and develop the ability to absorb workplace cultural norms. In order to effectively measure wellbeing of nurses and its changes, standardized scales measuring for instance job satisfaction and turnover intent (e.g., McCloskey/Mueller Satisfaction Scale) could be implemented. Nursing managers could also benefit from qualitative measures in the form of interviews or focus groups by acquiring in-depth knowledge about the difficulties experienced by nurses and ways of coping with them.

Finally, it should be mentioned that the study results refer to United Nations' Sustainable Development Goal 8 (SDG 8, Decent Work and Economic Growth) (4) as they present a valuable insight into how nurses' workplace wellbeing can be increased. Our study focused on a specific sample of healthcare personnel—in this way it also refers to SDG 3 (Good Health and WellBeing) (4) as the consequences of workplace wellbeing of this group reach the whole population whose health is highly dependent on their work.

## Conclusions

This study is one of the first to analyze work engagement among Polish nurses. Our main conclusion is that job resources, in the form of support from organization and community may be related to engagement directly, as well as indirectly through increased individual resources such as self-efficacy, ego-resiliency as well as job crafting behaviors. This highlights the importance of nurses' professional relationships, which determine their commitment and willingness to remain in the profession. If these relationships are beneficial, they contribute to the expansion of individual resources that influence the quality of care and translate into proactive changes in social relationships at work and the tasks performed. Therefore, it seems important to provide training for nursing leaders that emphasizes the proper treatment of subordinates by ensuring a sense of transparency, fairness, and appreciation for their efforts through appropriate rewards. On the other hand, training for nurses themselves, focusing on the ability to shape positive relationships with coworkers and a positive social climate at work, seems necessary.

In this context, the novel paradoxical finding that community support among nurses primarily operates through job crafting rather than through direct effects means that interventions aimed at increasing work engagement must enable active job crafting, not just provide passive support. Additionally, our contribution shows that the indirect effect of job crafting, but not personal resources, seems the most important for increasing work engagement among nurses.

## Data Availability

The raw data supporting the conclusions of this article will be made available by the authors, without undue reservation.

## References

[1] World Health Organization. State of the World's Nursing 2025 – Investing in Education, Jobs and Leadership. Geneva: World Health Organization (2025).

[2] Lesnik T. Hauser-Oppelmayer A. Turnover intention among intensive care nurses and the influence of the COVID-19 pandemic: a scoping review. Hum Resour Health. (2025) 23:23. doi: 10.1186/s12960-025-00992-7 40375274 PMC12080060

[3] Kim Y Han K. Longitudinal associations of nursing staff turnover with patient outcomes in long-term care hospitals in Korea. J Nurs Manag. (2018) 26:518–24. doi: 10.1111/jonm.12576 29318685

[4] United Nations. The 17 Goals. (2015). Available online at: https://sdgs.un.org/goals (Accessed February 15, 2026).

[5] Schaufeli W Salanova M Gonzalez-Roma V Bakker A. The measurement of engagement and burnout: a two sample confirmatory factor analytic approach. J Happiness Stud. (2002) 3:71–92. doi: 10.1023/A:1015630930326

[6] De Simone S Planta A Cicotto G. The role of job satisfaction, work engagement, self-efficacy and agentic capacities on nurses' turnover intention and patient satisfaction. Appl Nurs Res. (2018) 39:130–40. doi: 10.1016/j.apnr.2017.11.004 29422148

[7] Garcia-Sierra R Fernandez-Castro J Martinez-Zaragoza F. Work engagement in nursing: an integrative review of the literature. J Nurs Manag. (2016) 24:E101–11. doi: 10.1111/jonm.12312 26032875

[8] Keyko K Cummings GG Yonge O Wong CA. Work engagement in professional nursing practice: A systematic review. Int J Nurs Stud. (2016) 61:142–64. doi: 10.1016/j.ijnurstu.2016.06.003 27351831

[9] Bakker AB Demerouti E. The job demands–resources model: State of the art. J Manag Psychol. (2007) 22:309–28. doi: 10.1108/02683940710733115

[10] Brunetto Y Xerri M Shriberg A arr-Wharton R Shacklock K Newman S . The impact of workplace relationships on engagement, well-being, commitment and turnover for nurses in Australia and the USA. J Adv Nurs. (2013) 69:2786–799. doi: 10.1111/jan.12165 23651183

[11] Othman N Nasurdin AM. Social support and work engagement: a study of Malaysian nurses. J Nurs Manag. (2013) 21:1083–090. doi: 10.1111/j.1365-2834.2012.01448.x 23409702

[12] Broetje S Jenny GJ Bauer GF. The key job demands and resources of nursing staff: an integrative review of reviews. Front Psychol. (2020) 11:84. doi: 10.3389/fpsyg.2020.00084 32082226 PMC7005600

[13] Pohl S Battistelli A Djediat A Andela M. Emotional support at work: a key component for nurses' work engagement, their quality of care and their organizational citizenship behaviour. Int J Afr Nurs Sci. (2022) 16:100424. doi: 10.1016/j.ijans.2022.100424

[14] Tims M Bakker AB Derks D. The impact of job crafting on job demands, job resources, and well-being. J Occup Health Psychol. (2013) 18:230–40. doi: 10.1037/a0032141 23506549

[15] Hobfoll SE. Conservation of resources: a new attempt at conceptualizing stress. Am Psychol. (1989) 44:513–24. doi: 10.1037/0003-066X.44.3.513 2648906

[16] Wang X Liu L Zou F Hao J Wu H. Associations of occupational stressors, perceived organizational support, and psychological capital with work engagement among Chinese Female Nurses. BioMed Res Int. (2017) 2:1–11. doi: 10.1155/2017/5284628 28168198 PMC5266809

[17] Schwarzer R Jerusalem M. The general self-efficacy scale (GSE). Anxiety Stress Coping. (2010) 12:329–45.

[18] Block J Kremen AM. IQ and ego-resiliency: conceptual and empirical connections and separateness. J Personal Soc Psychol. (1996) 70:349. doi: 10.1037/0022-3514.70.2.349 8636887

[19] Suo R Zeng X Hao L Ye X Huang H Fan X . Relationships between job engagement and self-efficacy, perceived organisational support and perceived job security for newly recruited nurses: a cross-sectional study. J Adv Nurs. (2025) 1–10. doi: 10.1111/jan.70361. [Epub ahead of print]. 41191962

[20] Cao X Chen L. Relationships among social support, empathy, resilience and work engagement in haemodialysis nurses. Int Nurs Rev. (2019) 66:366–73. doi: 10.1111/inr.12516 31016716

[21] Pérez-Fuentes MDC Molero Jurado MDM Barragán Martín AB Simón Márquez MDM Martos Martínez Á Gázquez Linares JJ. The mediating role of perceived stress in the relationship of self-efficacy and work engagement in nurses. J Clin Med. (2018) 8:10. doi: 10.3390/jcm8010010 30577596 PMC6352085

[22] Pan X Mao T Zhang J Wang J Su P. Psychological capital mediates the association between nurses' practice environment and work engagement among Chinese male nurses. Int J Nurs Sci. (2017) 4:378–83. doi: 10.1016/j.ijnss.2017.09.009 31406781 PMC6626174

[23] Bakker AB Rodríguez-Muñoz A Sanz Vergel AI. Modeling job crafting behaviours: implications for work engagement. Hum Relat. (2016) 69:169–89. doi: 10.1177/0018726715581690

[24] Wrzesniewski A Dutton JE. Crafting a job: revisioning employees as active crafters of their work. Academy Manag Rev. (2001) 26:179–201. doi: 10.2307/259118

[25] Sheehan C Tham TL Holland P Cooper BK Newman A. The relationship between HIWPs and nurse work engagement: the role of job crafting and supervisor support. Int J Hum Res Manag. (2023) 34:1–27. doi: 10.1080/09585192.2021.1956564

[26] Cheng H Yang H Ding Y Wang B. Nurses' mental health and patient safety: an extension of the Job Demands–Resources model. J Nurs Manag. (2020) 28:653–63. doi: 10.1111/jonm.12971 32052511

[27] Park S Ha Y. The relationship between positive psychological capital and work engagement in clinical nurses: mediation effect of job crafting. BMC Nurs. (2025) 24:117. doi: 10.1186/s12912-024-02600-w 39901251 PMC11789409

[28] Lee K Seo JK Lee SE Wu KS. Job crafting as the missing link: understanding its role in nurses' work engagement. J Nurs Manag. (2025) 2025:e9420686. doi: 10.1155/jonm/9420686 41472869 PMC12745053

[29] Li JZ Lao Y Xing Y Shen Y Zhang M Xia Y . Exploring factors that affect job crafting behavior among nurses: a qualitative study. Nurs Ethics. (2026) 0:1–19. doi: 10.1177/09697330261418160 41778786

[30] Gadomska A. Koronawirus w czwartek 30 grudnia. Znów ponad 700 zgonów (2021). Available online at: https://biqdata.wyborcza.pl/biqdata/7,159116,27958270,koronawirus-w-czwartek-30-grudnia-nieoficjalnie-znow-ponad.html (Accessed April 14, 2026).

[31] Polish Radio. Poland reports new daily record of 53,420 COVID-19 cases. (2022). Available online at: https://www.polskieradio.pl/395/7784/Artykul/2890880,Poland-reports-new-daily-record-of-53420-COVID19-cases (Accessed March 20, 2026).

[32] Williams R. Why are there so few male nurses? (2017). Available online at: https://www.theguardian.com/healthcare-network/2017/mar/01/why-so-few-male-nurses (Accessed March 20, 2026).

[33] Naczelna Izba Pielegniarek I. Położnych (Polish Main Chamber of Nurses and Midwifes). Raport o stanie pielegniarstwa i położnictwa w Polsce. (2023).

[34] Szabowska-Walaszczyk A Zawadzka A Wojtaś M. Zaangażowanie w pracȩ i jego korelaty: adaptacja skali UWES autorstwa Schaufeliego i Bakkera. Psychol Jakości Życia. (2011) 10.

[35] Leiter MP Maslach C. Areas of worklife: a structured approach to organizational predictors of job burnout. In: Perrewé PL Ganster DC, editors. Emotional and Physiological Processes and Positive Intervention Strategies. Oxford: Elsevier Science (2004). p. 91–134. doi: 10.1016/S1479-3555(03)03003-8

[36] Terelak JF Izwantowska A. Adaptation of the areas of worklife survey C. Maslach and M Leitera Stud Psychol UKSW. (2009) 9:223–32.

[37] Eisenberger R Stinglhamber F. Perceived organizational support: Fostering enthusiastic and productive employees. Washington: American Psychological Association (2011). doi: 10.1037/12318-000

[38] Aryee S Srinivas ES Tan HH. Rhythms of life: antecedents and outcomes of work-family balance in employed parents. J Appl Psychol. (2005) 90:132–46. doi: 10.1037/0021-9010.90.1.132 15641894

[39] Schwarzer R Jerusalem M Juczyński Z. “Wersja polska GSES. Wydanie drugie” In: Juczyński Z, editor. Narzedzia pomiaru w promocji i psychologii zdrowia NPPPZ Warszawa: Pracownia Testów Psychologicznych PTP (2012).

[40] Kołodziej-Zaleska A Przybyła-Basista H. Ego-resiliency jako zasób osobisty – narzedzie pomiaru i jego wykorzystanie w badaniach interdyscyplinarnych. Czas Psychol – Psychol J. (2018) 24:159–70. doi: 10.14691/CPPJ.24.1.159

[41] Slemp GR Vella-Brodrick DA. The job crafting questionnaire: a new scale to measure the extent to which employees engage in job crafting. Int J wellbeing. (2013) 3:126–146. doi: 10.1037/t74913-000

[42] Kasprzak E Michalak M Minda M. The job crafting questionnaire: polish adaptation. Soc Psychol Bul. (2017) 43:459–75.

[43] Hayes AF. Beyond baron and kenny: statistical mediation analysis in the new millennium. Commun Monogr. (2009) 76:4: 408–20. doi: 10.1080/03637750903310360

[44] Lachowska B Minda K. Burnout and work engagement among psychiatric nurses – are work characteristics important? Arch Psych Psych. (2020) 1:77–83. doi: 10.12740/APP/113487

[45] Aydogdu ALF. Work engagement among nurses in the context of the COVID-19 pandemic: A systematic review. Nurs Ethics (2024) 31:1688–708. doi: 10.1177/09697330241257570 38835110

[46] Wang Y Tang L. and Li L. Work engagement and associated factors among healthcare professionals in the post-pandemic era: a cross-sectional study. Front Public Health. (2023) 11:1173117. doi: 10.3389/fpubh.2023.1173117 37575106 PMC10413104

[47] Aungsuroch Y Gunawan J Junamasta I.G. Montayre J. Updating factors influencing nurse work engagement in the hospital settings: a systematic review. J Healthc Leadersh. (2024) 16:157–176. doi: 10.2147/JHL.S451056 38523801 PMC10961065

[48] Nasurdin AM Ling TC Khan SN. Linking social support, work engagement and job performance in nursing. Int J Bus Soc. (2018) 19:363–386.

[49] Mehrad A Fernández-Castro J Pau González Gómez de Olmedo M García-Sierra R. Mediation role of perceived organizational support on nurses' work engagement and leadership styles. (2022). Available online at: https://ejournal.undip.ac.id/index.php/medianers/article/download/45872/22136 (Accessed April 17, 2026).

[50] Blau P. Exchange and Power in Social Life. Wiley: New York (1964).

[51] Nantsupawat A Kutney-Lee A Abhicharttibutra K Wichaikhum OA Poghosyan L. Exploring the relationships between resilience, burnout, work engagement, and intention to leave among nurses in the context of the COVID-19 pandemic: a cross-sectional study. BMC Nurs. (2024) 23:290. doi: 10.1186/s12912-024-01958-1 38685024 PMC11057140

[52] Xanthopoulou D Bakker AB Demoerouti E Shaufeli WB. The role of personal resources in the job demands-resources model. Int J Stress Manag. (2007) 14:121–41. doi: 10.1037/1072-5245.14.2.121

[53] Lin H Deng C Zhuoxi X. Positive mental health literacy and job well-being among palliative care nurses: the mediating effect of job crafting. BMC Psychol. (2025) 13:949. doi: 10.1186/s40359-025-03206-w 40836263 PMC12369148

[54] Ryan RM Deci EL. Self-determination theory and the facilitation of intrinsic motivation, social development, and well-being. Am Psychol. (2000) 55:68–78. doi: 10.1037/0003-066X.55.1.68 11392867

[55] Tims M Parker SK. How coworkers attribute, react to, and shape job crafting. Organ Psychol Rev. (2020) 10:29–54. doi: 10.1177/2041386619896087

[56] Shalev D Traeger LN Doyle K Kiser SB Brenner KO Rosenberg LB . Turning the lens inward: the psychological elements of clinician well being. J Palliat Med. (2022) 25:349–54. doi: 10.1089/jpm.2021.0548 35085468 PMC10331146

[57] Ibrahim AM Zaghamir DEF Elsehrawy MG Abdel-Aziz HR Elgazzar SE Hassabelnaby FGE . Impact of job crafting and work engagement on the mental and physical health of palliative care nurses. BMC Nurs. (2025) 24:404. doi: 10.1186/s12912-025-03058-0 40211286 PMC11987278

[58] Ojala H Kuivila H-M Mikkonen K Jarva E Juntunen J. Factors associated with newly graduated nurses' work engagement: systematic review of quantitative studies. J Adv Nurs. (2026) 82:1947–972. doi: 10.1111/jan.17069 40576017 PMC12907590

